# Supplementary material for: Oncolytic H-1 Parvovirus Enters Cancer Cells through Clathrin-Mediated Endocytosis
Source: Viruses. 2020 Oct 21;12(10):1199. doi: 10.3390/v12101199 (PMC7594094; doi:10.3390/v12101199)
Supplement: Supplementary file 1 [file viruses-12-01199-s001.zip › Supplementary.docx]

**
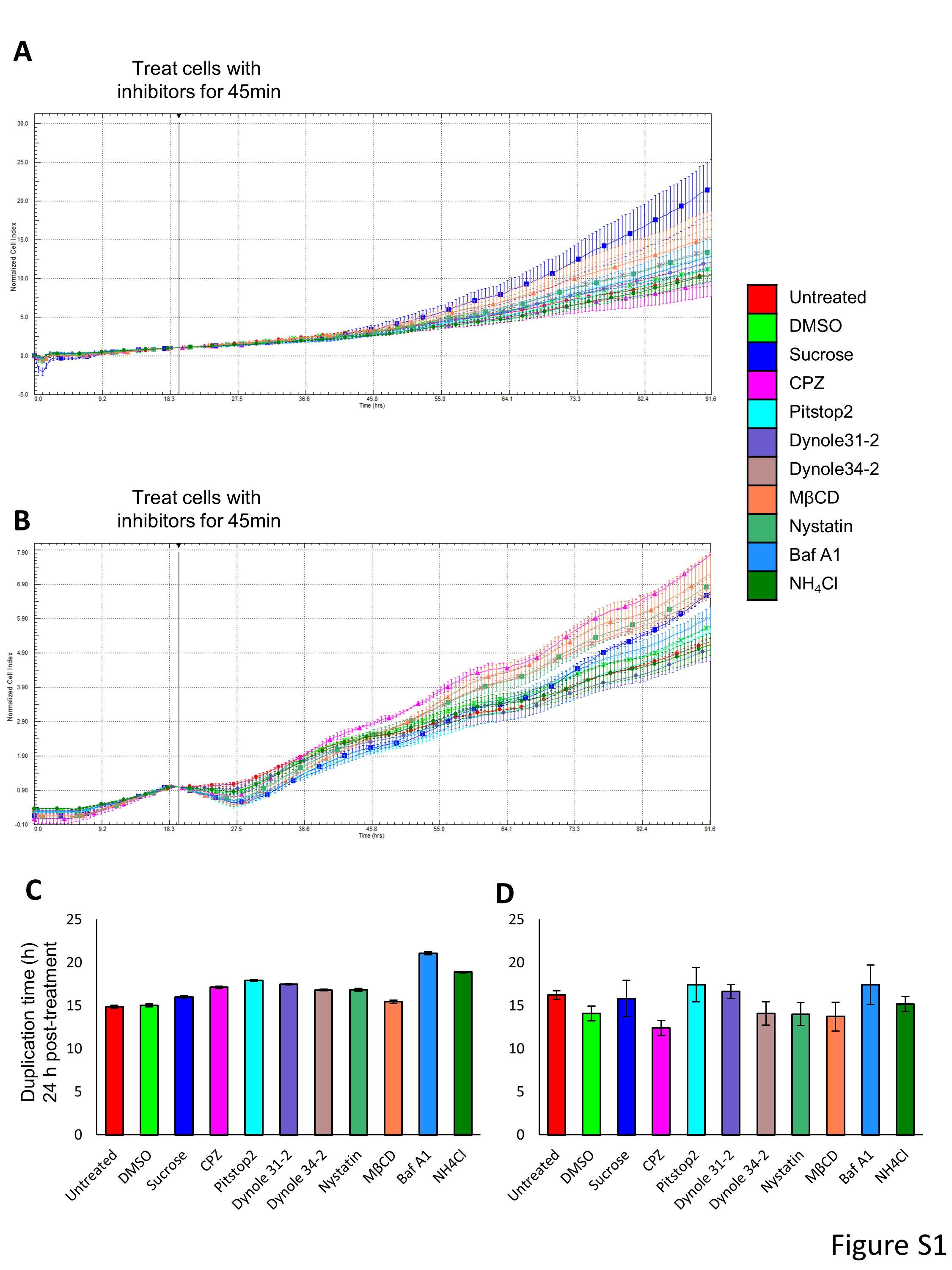
**

**Figure S1.** Entry pathway inhibitors did not alter cell proliferation. **(A)** HeLa and **(B)** NCH125 cells were seeded in a 96-well E-plate and left untreated or treated with DMSO, hypertonic sucrose, chlorpromazine (CPZ), pitstop 2, Dynole 31-2, Dynole 34-2, MβCD, nystatin, bafilomycin A1 (BafA1) or ammonium chloride (NH_4_Cl) for 45 min and then grown for an additional 72 hours. Inhibitors were applied at the same concentrations used for studying H-1PV entry pathways. Cell growth was monitored with the xCELLigence System. Curves represent the mean Cell Index value from 3 wells ± standard deviation (n=3). **(C)** HeLa or **(D)** NCH125 cells’ doubling time for the first 24 hours after treatment was generated using the xCELLigence System. Data is plotted as the mean Cell Index value from 3 wells ± standard deviation.

**
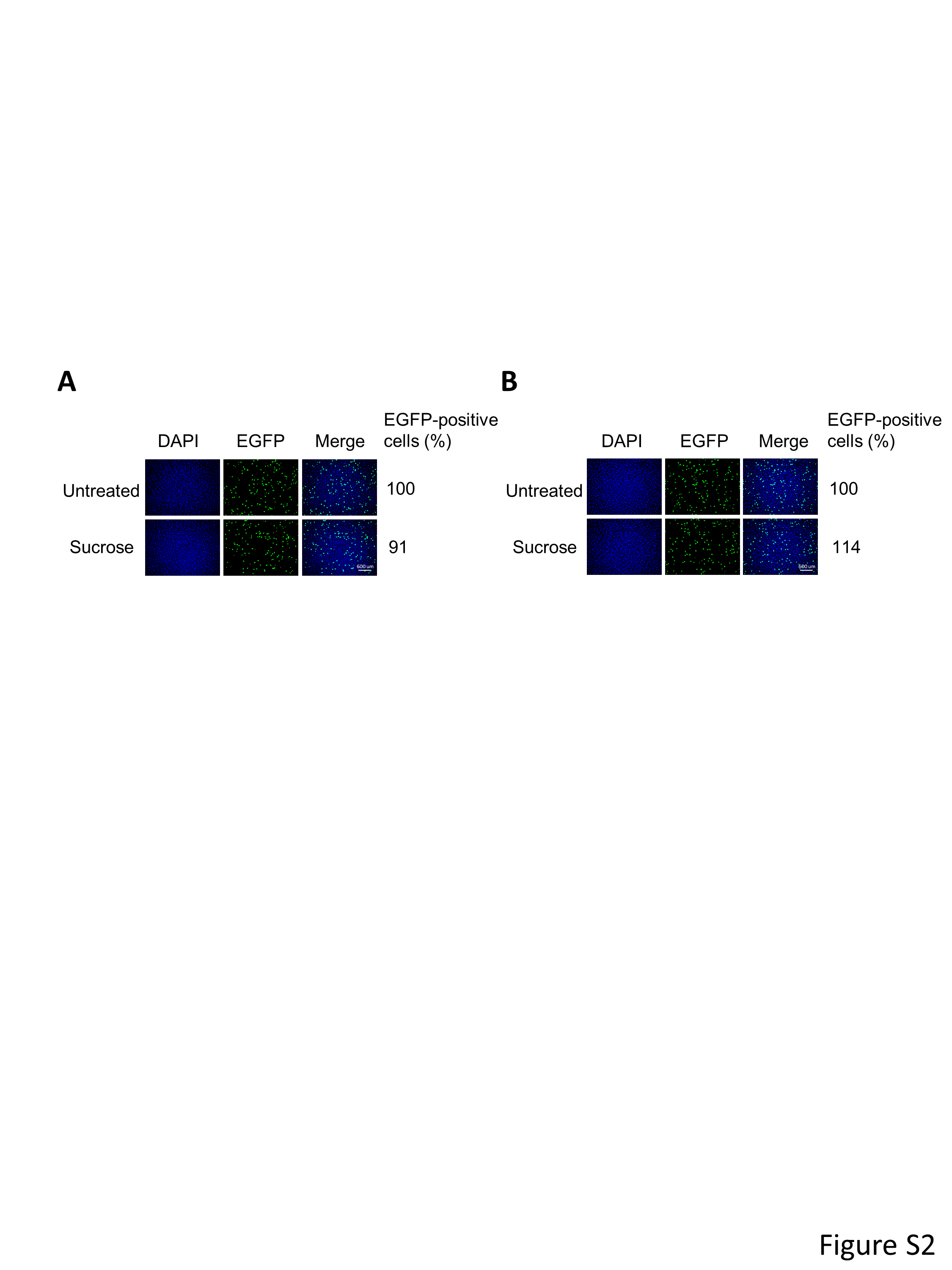
**

**Figure S2.** Sucrose, a classical inhibitor of clathrin-mediated endocytosis, has no impact on virus transduction if added 4 hours after virus infection. **(A)** HeLa and **(B)** NCH125 cells were infected with recH-1PV-EGFP for 4 hours, and then treated or not with sucrose for 45 min. At 20 hours post-infection, cells were processed as described in the M&M section. Numbers represent the average percentage of EGFP-positive cells relative to the number of EGFP-positive cells observed in untreated cells, which was arbitrarily set as 100%.
